# A Novel Two-Component Response Regulator Links *rpf* with Biofilm Formation and Virulence of *Xanthomonas axonopodis* pv. *citri*

**DOI:** 10.1371/journal.pone.0062824

**Published:** 2013-04-23

**Authors:** Tzu-Pi Huang, Kuan-Min Lu, Yu-Hsuan Chen

**Affiliations:** Department of Plant Pathology, National Chung-Hsing University, Taichung, Taiwan; Centre National de la Recherche Scientifique, Aix-Marseille Université, France

## Abstract

Citrus bacterial canker caused by *Xanthomonas axonopodis* pv. *citri* is a serious disease that impacts citrus production worldwide, and *X. axonopodis* pv. *citri* is listed as a quarantine pest in certain countries. Biofilm formation is important for the successful development of a pathogenic relationship between various bacteria and their host(s). To understand the mechanisms of biofilm formation by *X. axonopodis* pv. *citri* strain XW19, the strain was subjected to transposon mutagenesis. One mutant with a mutation in a two-component response regulator gene that was deficient in biofilm formation on a polystyrene microplate was selected for further study. The protein was designated as BfdR for *b*iofilm *f*ormation *d*efective *r*egulator. BfdR from strain XW19 shares 100% amino acid sequence identity with XAC1284 of *X. axonopodis* pv. *citri* strain 306 and 30–100% identity with two-component response regulators in various pathogens and environmental microorganisms. The *bfdR* mutant strain exhibited significantly decreased biofilm formation on the leaf surfaces of Mexican lime compared with the wild type strain. The *bfdR* mutant was also compromised in its ability to cause canker lesions. The wild-type phenotype was restored by providing pbfdR *in trans* in the *bfdR* mutant. Our data indicated that BfdR did not regulate the production of virulence-related extracellular enzymes including amylase, lipase, protease, and lecithinase or the expression of *hrpG, rfbC,* and *katE*; however, BfdR controlled the expression of *rpfF* in XVM2 medium, which mimics cytoplasmic fluids *in planta.* In conclusion, biofilm formation on leaf surfaces of citrus is important for canker development in *X. axonopodis* pv. *citri* XW19. The process is controlled by the two-component response regulator BfdR via regulation of *rpfF*, which is required for the biosynthesis of a diffusible signal factor.

## Introduction

*Xanthomonas axonopodis* pv. *citri* (syn. *X. citri* subsp. *citri*) affects most commercial citrus cultivars and causes citrus bacterial canker resulting in significant crop losses worldwide [1]. The bacteria are presumably considered as epiphytes on the plant surface before infection [2]. They infect leaves, stems, thorns and fruits and enter the citrus plant mainly through wounds and stomata [3]. Biofilms are formed when microorganisms attach to a surface and form a self-produced matrix of exopolysaccharides (EPS) that they embed themselves within [4]. Rigano *et al.* (2007) showed that biofilm formation is necessary for epiphytic fitness and canker development in *X. axonopodis* pv. *citri* [5]. Our previous study indicated that *Bacillus subtilis* TKS1-1 and *Bacillus amyloliquefaciens* WG6-14 can interfere with phyllosphere biofilm formation by *X. axonopodis* pv. *citri*, which may contribute to the attenuation of citrus bacterial canker symptom development [6]. These results and findings from other plant-associated bacteria suggest that biofilm formation plays important roles in pathogenesis [5], [6], [7], [8]. Thus, experiments were conducted to uncover genes and gene clusters that are involved in biofilm formation and virulence as well as to reveal potential interactions between these two processes in *X. axonopodis* pv. *citri*, which may aid in the development of a strategy for disease management.

Several approaches have been used to investigate and understand genes and gene clusters that govern mechanisms of pathogenesis and biofilm formation. These include comparative genome analyses, functional studies performed by mapping insertion sites within transposon-based mutant libraries [9], [10], construction and use of macroarrays [11], and directed mutagenesis of genes that encode proteins with specific functions [12], [13], [14], [15], [16]. Comparative genome analysis of *Xanthomonas campestris* pv. *campestris*, which causes black rot of crucifers, and *X. axonopodis* pv. *citri* revealed that more than 80% of the genes are shared between the two species; however, subsets of genes are strain-specific and may be responsible for distinct host specificity and pathogenicity. These genes include *avr*, genes encoding members of the type III secretion system, *rpf* (*r*egulation of *p*athogenicity *f*actors), genes encoding type IV fimbriae, and lipopolysaccharide (LPS) O-antigen operons [17]. In a functional study, Gottig *et al.* (2010) suggested that *X. axonopodis* pv. *citri* uses several mechanisms to colonize and invade its host [18]. Citrus canker bacteria could attach to the host surface via adhesins such as the filamentous hemagglutinin-like protein FhaB [13]. The bacteria may inject pathogenicity effectors into the host through the type III secretion system and modulate the plant's defense mechanisms [19], [20]. To prolong survival and colonization of the host, the bacteria form biofilms by producing EPS, xanthan which is synthesized by the *gum* operon [14], and FhaB [13]. Additionally, a single flagellum of *X. axonopodis* pv. *citri* was involved in the formation of mushroom-shaped structures in mature biofilms [15]. It was also found that the bacteria use a plant natriuretic peptide-like protein to modulate host homeostasis and cause the opening of stomata, an increase in photosynthesis and suppression of the plant's defense mechanisms to create favorable conditions for their survival [12]. Several factors were shown to be relevant for host colonization or biofilm formation of *X. axonopodis* pv. *citri*. These include the ColS/ColR two-component system (TCS) [21], filamentous-like adhesin [13], flagellin [15], UTP-glucose-1-phosphate uridylyltransferase [22], xanthan [5], [14], LPS synthesized by *wxacO* and *rbfC* [23], the photosensory protein Lov (light, oxygen, voltage) [24], the haloacid dehalogenase-like phosphatase XAC0482, and the two-component sensor RbfS [25]. Of the identified genes associated with biofilm formation, several were also thought to be involved in pathogenesis. Although an array of genes are involved in biofilm formation and/or virulence in *X. axonopodis* pv. *citri*, the regulatory network of these genes is relatively under-investigated.

The *d*iffusible *s*ignal *f*actor (DSF)-mediated cell-to-cell communication system [15], [16] was first identified in *X. campestris* pv. *campestris*, and components of this system were shown to be synthesized and regulated by the *rpf* gene cluster [26]. *rpfB* and *rpfF* are responsible for the synthesis of the DSF cis-Δ2-11-methyl-dodecanoic acid, and the *rpfGHC* operon encodes for a TCS that is responsible for regulation [26]. Similar to the findings in *X. campestris* pv. *campestris*, the *rpf*/DSF system in *X. axonopodis* pv. *citri* was shown to regulate virulence factors such as extracellular cyclic β-(1,2)-glucan; proteases; endoglucanases; genes involved in flagella-dependent and independent motility, chemotaxis, and flagellar biosynthesis; genes involved in the TCA cycle and in the degradation of celluloses and glucans; the transcription factor σ54; and genes encoding hypothetical proteins [15], [16], [27]. Four of the hypothetical proteins share a high level of identity with XagA, XagB, XagC and XagD of *X. campestris* pv. *campestris*, which were found to be involved in biofilm formation and may contribute to adhesins biosynthesis [27]. Mutations in *X. axonopodis* pv. *citri rpfF*, *rpfG* and *rpfC* caused a reduction in bacterial attachment to grapefruit leaves and to abiotic surfaces in either XVM2 media or nutrient broth [27], which is in contrast to the findings in *X. campestris* pv. *campestris*. The *rpfG* and *rpfGHC* mutants of *X. campestris* pv. *campestris* showed increased initial attachment to plastic surfaces compared with the wild type [28]. By DNA microarray analysis of the RpfF, RpfG and RpfC regulons in *X. axonopodis* pv. *citri*, Guo *et al.* (2012) found a conserved group of genes that were regulated by all three proteins, suggesting a major role for RpfG and RpfC in the perception and transduction of signals in the *rpf*/DSF system [27]. However, some genes were controlled by only one of the three proteins, suggesting that the RpfG and RpfC TCS may regulate additional genes beyond those involved in the transduction of the DSF signal [27].

To understand the regulatory mechanisms of biofilm formation by *X. axonopodis* pv. *citri*, we subjected strain XW19 to transposon mutagenesis. One individual with a mutation in a two-component response regulator was identified that exhibited a defect in biofilm formation on polystyrene plates and on the leaf surfaces of citrus plants. Thus, the identified response regulator was designated as BfdR for *b*iofilm *f*ormation *d*efective *r*egulator, and its flanking two-component *s*ensor was designated as BfdS. We also provide evidence that BfdR is involved in the pathogenesis and regulation of the *rpf*/DSF system.

## Materials and Methods

### Bacterial strains and plant growth conditions

The *Xanthomonas* and *Escherichia coli* strains and plasmids used in this study are listed in Table 1. *Xanthomonas* strains were routinely cultured on Tryticase^TM^ Soy (TS) agar or in TS broth (Becton Dickinson, Franklin Lakes, NJ, USA) at 27°C unless otherwise stated. All *E. coli* strains were grown in Luria-Bertani (LB) broth (Becton Dickinson) at 37°C. When required, the medium was supplemented with gentamicin (Gm; Sigma-Aldrich, St. Louis, MO, USA; 10 μg/ml for *Xanthomonas* strains and 25 μg/ml for *E. coli*) or kanamycin (Km; Sigma-Aldrich; 50 μg/ml).

**Table 1:** Bacterial strains and plasmids used in this study.

Strains and Plasmids	Relevant characteristics	Source
**Strains**		
***E. coli***		
DH5α	λ^−^ φ80d*lac*ZΔ*M15* Δ(*lacZYA*-*argF*)*U169 recA1 endA1 hsdR17*(r_K_^−^ m_K_^−^) *phoA supE44 thi*-*1 gyrA96 relA1*	Invitrogen
***X. axonopodis pv. citri***		
XW19	Wild type	[73]
TPH1	Km^r^, two-component response regulator::EZ-TN transposon mutant, XW19 derivative	This study
TPH2	Gm^r^, XW19 harboring pBBR1MCS5	This study
TPH3	Gm^r^, TPH1 harboring pBBR1MCS5	This study
TPH4	Gm^r^, TPH1 harboring pbfdSR	This study
TPH5	Gm^r^, TPH1 harboring pbfdR	This study
**Plasmids**		
pBBR1MCS5	Gm^r^, broad-host range cloning vector	[74]
pGTKan	Gm^r^, 131 bp *nptII* promoter driven *gfp*	[75]
pbfdSR	Gm^r^, 1954 bp promoters and coding regions of two-component sensor and response regulator	This study
pbfdR	Gm^r^, 696 bp promoter and coding region of two-component response regulator	This study

The citrus plants used in the study included navel orange (*Citrus sinensis* [L.] Osbeck), Mexican lime (*Citrus aurantifolia* [C.] Swingle) and Ruby grapefruit (*Citrus × paradise* Macfad). The navel orange was grafted onto a Cantonese lemon (*C. limonia*), which was used as the rootstock, whereas the Mexican lime was grafted onto a Swingle citrumelo. The plants were cultivated in potting mix (nacrite:vermiculite:loam:organic compost  = 1∶0.8∶3∶0.48) in 60 cm diameter pots and maintained in a greenhouse. For the pathogenicity assay, 30-day-old newly grown leaves were used.

### Generation of transposon mutants and sequence analysis of inserted genes

*X. axonopodis* pv. *citri* strain XW19 transposon mutants were generated using EZ::TN <R6Kγori/KAN-2> Tnp Transposome (Epicentre, Madison, WI, USA) as described by Huang *et al*. [29]. The transposon flanking regions were “rescue cloned” as described by the manufacturer and sequenced using the primers KAN-2 FP-1 and R6KAN-2 RP-1 (Table 2). Additional primers were used to fully sequence the genes and flanking regions. DNA sequencing was performed at the Automated DNA Sequencing Service Laboratory, National Chung-Hsing University, Taiwan. The sequences were compared with those in the GenBank nucleotide database using the BLAST program (http://www.ncbi.nlm.nih.gov/BLAST). The nucleotide sequences were translated into amino acid sequences using the ExPASy translate tool (Bioinformatics Resource Portal, http://web.expasy.org/translate/SwissInstitateofBioinformatics) and compared with those in the GenBank database. The percent identity of protein sequences was analyzed using the FASTA program (Ver. 36.3.6) at the University of Virginia [30]. The amino acid sequences were aligned using the Pileup program, SeqWeb version 3.1.2 (GCG Wisconsin Package, Accelrys Inc., San Diego, CA, USA). The conserved domains were analyzed using the NCBI Conserved Domain Assay (National Center for Biotechnology Information, http://www.ncbi.nlm.nih.gov/) and the simplified molecular architecture research tool (SMART) [31].

**Table 2:** Primers used in this study.

Gene	Primer sequence (5′-3′)^a^	Protein/Source
**Sequencing & complementation primers**		
KpnI-4-3D-F1	F:CGGGGTACCGCCAATGCTGCGATTGACCGAG	Two-component sensor and response regulator
KpnI-4-3D-F2	F:CGGGGTACCAATAGTTGCGATGCGATGCCCTCT	Two-component response regulator
KpnI-4-3D-R	R:CGGGGTACCCTACGCGTTGGCTGGGGTGGCCTTGAGC	
KAN-2 FP-1	ACCTACAACAAAGCTCTCATCAACC	Epicentre
R6KAN-2 RP-1	CTACCCTGTGGAACACCTACATCT	Epicentre
**RT-PCR primers**		
*hrpG*	F:GCCTTTCAATTCGCACGAGTTACACG R:CACACGCCGGGGCTGGAAAAGA	TTSS component regulator
*katE*	F:TCAATGAGAAAGGCGAGAGCACCT R:AGATCGCGACGGTGAAAGTCTTGA	Monofunctional catalase [21]
*rfbC*	F:ATCCATCACCAGCACCTGTTCGTA R:GAATCCGCCAATGGCATCGAAGTT	LPS O-antigen biosynthesis protein [21]
*rpfF*	F:ATGAACACGATTGAAAAGATTTCCCTCG R:TCAGGCGACGCCCATGCCGACGCGC	Regulation of pathogenicity factor and DSF biosynthesis
*rpoD*	F:CATTCCAGGTTGGTCTGGTT	Sigma factor σ^70^
	R:TACGCCAAGTTCAAGAAGGT	

a F, forward primer; R, reverse primer; underline, restriction enzyme sites.

### Construction of plasmids and strains

**Figure 1:**
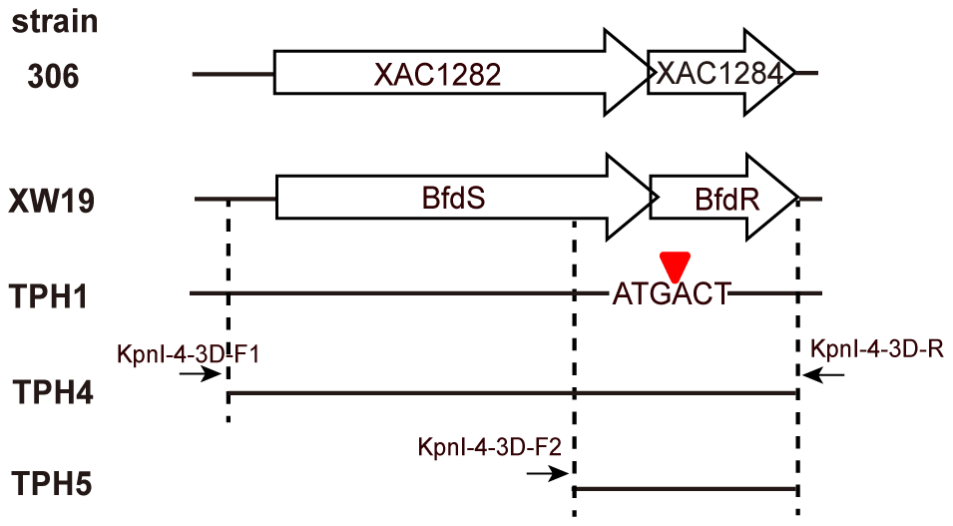
Schematic diagram of *bfdS* and *bdfR* and their homologues in *X. axonopodis* pv. *citri* strains XW19 and 306. The open arrows show the locations and orientations of the genes. The position of EZ-Tn*5* in the mutant is indicated by an inverted red triangle. The construction of the complementation plasmids pbfdSR and pbfdR is described in Materials and Methods. The primers used to construct the plasmids for complementation are shown on the top of the solid arrows.

To construct the plasmids for complementation, the *bfdR* and *bfdS* ribosomal binding sites, native promoters and coding regions were amplified from *X. axonopodis* pv. *citri* strain XW19 by PCR using the primers KpnI-4-3D-F1 and KpnI-4-3D-R; and *bfdR* ribosomal binding site, and the native promoter and coding region were amplified using the primers KpnI-4-3D-F2 and KpnI-4-3D-R (Table 2 and Figure 1). The products were subsequently cloned into pGEM-T Easy (Promega, Madison, WI). The fragment containing *bfdS* and *bfdR* (1954 bp) was excised with *Kpn*I and ligated into pBBR1MCS5 to generate pbfdSR, whereas *bfdR* (696 bp) was excised with *Kpn*I and ligated into pBBR1MCS5 to generate pbfdR. For complementation, pbfdSR or pbfdR was electroporated (12.5 kV/cm, 25 μF, 400 Ω) into the *bfdR* mutant (TPH1). Electroporation, restriction endonuclease digestion, PCR, cloning, DNA extraction, and DNA purification were performed using standard procedures [32]. For the biofilm and pathogenicity assays, *X. axonopodis* pv. *citri* strains TPH2 and TPH3 were generated by electroporating pBBR1MCS5 into *X. axonopodis* pv. *citri* strain XW19 and strain TPH1, respectively. For confocal laser scanning microscopy, pGTKan was electroporated into *X. axonopodis* pv. *citri* strains TPH2, TPH3 and TPH5.

### Biofilm formation assay

Transposon mutants were screened for biofilm formation using a microplate assay adapted from Fletcher (1997) and O'Toole *et al*. (1993) [33], [34]. Briefly, wells containing 2 ml of TS broth supplemented with 50 μg/ml kanamycin were inoculated with overnight bacterial cultures to an optical density at 620 nm (OD_620_) of 0.05 and incubated at 27°C with shaking (50 rpm) for 2 days. Biofilm cells were stained with 0.1% crystal violet and washed. Subsequently, the stain remaining in the cells was solubilized with 70% ethanol, and the OD_620_ was determined using a Tecan Infinite M200 plate reader (Tecan Austria GmbH, Grödig, Austria). The mutant TPH1 grew similarly to wild type but was deficient in biofilm formation; thus, TPH1 was selected for further characterization.

### Epifluorescence and confocal laser scanning microscopy

*X. axonopodis* pv. *citri* strains TPH2, TPH3 and TPH5 harboring pGTKan were cultured with leaf discs obtained from grapefruit, Mexican lime and navel orange plants in a 24-well polystyrene microplate under conditions similar to those used for the biofilm formation assay. For epifluorescence microscopy, cells colonized on the leaf surfaces were examined with a Leica DMLB microscope (Leica, Wetzlar, Germany) equipped with an XF100-2 filter. The excitation and emission wavelengths were 475 nm and 535 nm, respectively. Digital images were acquired using an AxioCamHRc camera (Carl Zeiss, Jena, Germany) and analyzed using AxioVision software (Carl Zeiss). For confocal laser scanning microscopy, an Olympus Fluoview FV1000 confocal microscope (Olympus Optical Co. Ltd., Tokyo, Japan) equipped with an argon laser was used. The excitation and emission wavelengths were 510 nm and 488 nm, respectively.

### Epiphytic growth and pathogenicity assay

*X. axonopodis* pv. *citri* strains TPH2, TPH3 and TPH5 were cultured in TS broth supplemented with 10 μg/ml gentamicin at 27°C with shaking at 100 rpm for 2 days. The culture suspensions were adjusted to an OD_620_ of 0.3 (1×10^8^ cfu/ml) and subsequently sprayed on the leaves of Mexican lime (20 leaves per strain) to the point of runoff in the greenhouse. *In planta* growth assays were performed by grinding 0.5-cm diameter leaf discs (20 discs per leaf) from artificially inoculated leaves (3 leaves per strain) in 1 ml of sterile Milli-Q water followed by serial dilutions and plating onto *Xanthomonas* differential (Xan-D) medium [35]. Leaves were collected at 0, 1, 2, 4, and 6 days post-inoculation. Colonies were counted after 2 days of incubation at 27°C, and the results are presented as log cfu/cm^2^ of leaf tissue. The same culture and inoculation conditions used for the *in planta* growth assays were used for the pathogenicity assay. At two months post-inoculation, cankers were counted on six leaves inoculated with different strains, and the areas of the counted leaves were measured on digital images using Adobe Photoshop software (Adobe Systems Inc, San Jose, CA, USA). The disease severity of citrus bacterial canker caused by the different strains was expressed as the number of cankers per cm^2^.

### Reverse transcription-PCR (RT-PCR) analysis of virulence-related genes

*X. axonopodis* pv. *citri* strains TPH2, TPH3 and TPH5 were cultured in TS broth and XVM2 medium [11] at 27°C for 2 days. The culture suspensions were diluted with media to an OD_620_ of 0.05 and incubated at 27°C with shaking at 100 rpm. Bacterial cells were collected after an 18 hr incubation period and subjected to RNA extraction using TRI Reagent® LS RNA Isolation Reagent (Molecular Research Center Inc, Cincinnati, OH, USA) according to the manufacturer's instructions. Contaminating genomic DNA was removed using the TURBO DNA-*free* ^TM^ Kit (Ambion, Austin, TX, USA). The RNA concentration was determined by measuring the absorbance at 260 nm with a Tecan Infinite M200 plate reader and adjusted to a concentration of 50 ng/ml. RT-PCR was performed with the Transcriptor One-Step RT-PCR Kit (Roche Applied Science, Indianapolis, IN, USA) in a 20 µl reaction mixture containing 50 ng of total RNA. Gene-specific primers (Table 2) were used for amplification of the virulence-related genes *rfbC* (113 bp), *hrpG* (747 bp), *rpfF* (870 bp), and *katE* (127 bp). The *rpoD* (264 bp) gene encoding sigma factor σ^70^ [36] was used as a loading control.

### Activity of extracellular enzymes

*X. axonopodis* pv. *citri* strains TPH2, TPH3, TPH4, and TPH5 were cultured in TS broth supplemented with 10 μg/ml Gm at 27°C with shaking at 100 rpm for 2 days. The culture suspension was diluted with TS broth to an OD_620_ of 0.05, and 10 μg of the diluted bacterial suspension was spotted onto starch agar for the detection of amylase [37], medium containing Tween 80 and skim milk for the detection of lipase and protease [35], and egg yolk agar for the detection of lecithinase [38]. The plates were incubated at 27°C for 2 days.

### Statistical analysis

All experiments were performed at least three times. The data are presented as means and standard deviations obtained from at least four replicates of a single representative experiment. The significant difference between the treatments was analyzed by one-way ANOVA and Tukey's honestly significant difference (HSD) test using SPSS 15.0 software (SPSS Inc, Chicago, IL, USA).

### Nucleotide sequence accession numbers

The *rpfF* and *bfdSR* sequences of *X. axonopodis* pv. *citri* strain XW19 were deposited in the GenBank database (accession numbers JX987963 and JX987964).

## Results

### The transposon-inserted gene is homologous to a gene encoding a two-component response regulator

Biofilm formation by plant-associated bacteria was shown to be important for pathogenesis or symbiosis [5], [7]. To understand the molecular mechanisms of biofilm formation and to elucidate the role of biofilm formation in symptom development, we subjected *X. axonopodis* pv. *citri* strain XW19 to transposon mutagenesis using the EZ-Tn5^TM^ <R6Kγori/KAN-2>Tnp Transposome^TM^ Kit. A total of 1710 transposon mutants were screened for defects in biofilm formation in 24-well polystyrene plates. One mutant had a growth rate that was similar to wild type in TS broth but was defective in biofilm formation. This mutant, TPH1, was selected for further investigation (data not shown). The transposon flanking regions were rescued by “rescue cloning” as described by the manufacturer (Epicentre) and sequenced using the primers KAN-2 FP-1 and R6KAN-2 RP-1 (Table 2). The transposon-inserted gene was homologous to the locus tag XAC1284, which encodes for a two-component response regulator in *X. axonopodis* pv. *citri* strain 306 (GenBank accession no. NC_003919). The flanking sequences of the transposon-inserted genes were homologous to the locus tag XAC1282, which encodes for the two-component sensor (Figure 1). Both of the genes in strain XW19 share 100% identity with those of strain 306 (data not shown).

**Figure 2:**
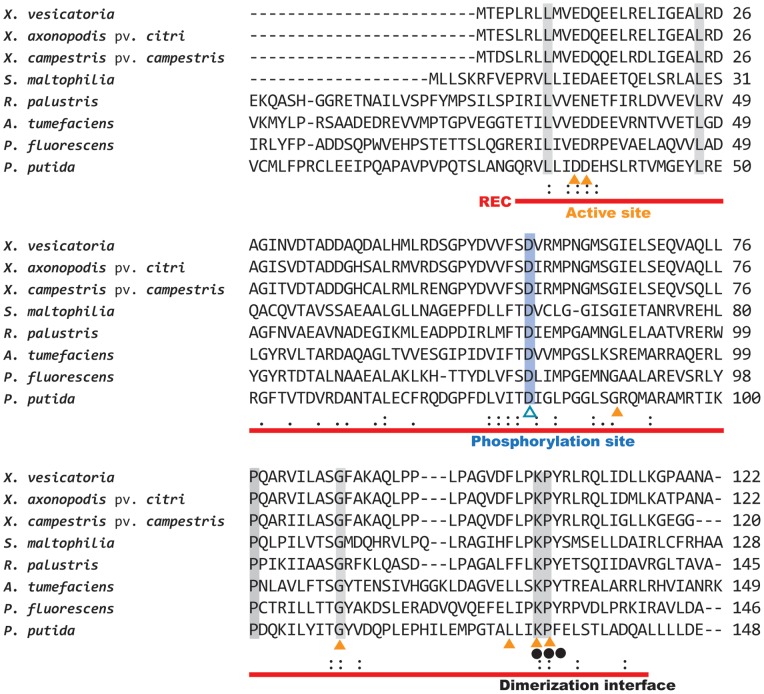
Alignment of the *Xanthomonas axonopodis* pv. *citri* XW19 two-component response regulator with its homologues in various organisms The putative signal receiver domain (REC) of the protein is depicted with a red line. Active sites (solid triangles) are present at amino acid (a.a.) positions 11, 12, 65, 85, 101,104, and 105 in *X. axonopodis* pv. *citri*; a phosphorylation site (open triangle) is present at a.a. position 56; and the dimerization interface (solid circle) is located at a.a. positions 104, 105, and 106. Identical (shading), highly conserved (:) and less conserved (.) a.a. residues are indicated. The GenBank accession number of the two-component response regulator homologue in *Xanthomonas vesicatoria* is ZP_08177161.1; that in *Xanthomonas campestris* pv. *campestris* is NP_636561.1; that in *Rhodopseudomonas palustris* is YP_567467.1; that in *Stenotrophomonas maltophilia* is YP_002029492.1; that in *Agrobacterium tumefaciens* is NP_356752.2; that in *Pseudomonas fluorescens* is ZP_07777428.1; and that in *Pseudomonas putida* is YP_001749312.1.

The nucleotide sequence of the transposon-inserted gene was translated into an amino acid sequence using the ExPASy translate tool. The translated amino acid sequence in *X. axonopodis* pv. *citri* strain XW19 was compared with sequences in *Xanthomonas vesicatoria*, *X. campestris* pv. *campestris*, *Agrobacterium tumefaciens*, *Pseudomonas putida*, *Pseudomonas fluorescens*, *Stenotrophomonas maltophilia* and *Rhodopseudomonas palustris*. An alignment of the amino acid sequences is shown in Figure 2. The amino acid sequence of BfdR in *X. axonopodis* pv. *citri* strain XW19 shares 89.3% identity with locus tag XVE_1034, which is a response regulator in *X. vesicatoria* strain ATCC 35937 (GenBank accession no. ZP_08177161) with a CheY-like receiver domain, ATPase domain, and DNA-binding domain. BfdR also shares 89.7% identity with locus tag XCC1187, a two-component response regulator in *X. campestris* pv. *campestris* strain ATCC33913 (GenBank accession no. NP_636561); 37.3% identity with locus tag PFWH6_4865, a response regulator in *P. fluorescens* WH6 (GenBank accession no. ZP_07777428) that harbors a receiver domain; 30.8% identity with locus tag PputW619_2443, a PAS/PAC sensor hybrid histidine kinase in *P. putida* strain W619 (GenBank accession no. YP_001749312); 35.1% identity with locus tag Atu3883, a chemotaxis response regulator in *A. tumefaciens* strain C58 (GenBank accession no. NP_356752); 35.7% identity with locus tag Smal_3110, a response regulator receiver protein in *S. maltophilia* strain R551-3 (GenBank accession no. YP_002029492); and 35.6% identity with locus tag RPD_0328, a response regulator receiver in *R. palustris* strain BisB5 (GenBank accession no. YP_567467) (data not shown). According to the conserved domain assay, the amino acid sequence of the transposon-inserted gene in *X*. *axonopodis* pv. *citri* strain XW19 contained a highly conserved CheY-homologous receiver domain, REC [39] (Figure 2). The REC domain in strain XW19 contains active sites at positions 11, 12, 65, 85, 101, 104, and 105; a phosphorylation site at position 56; and intermolecular recognition sites and dimerization interface sites at positions 104, 105, and 106. The active sites coordinate Mg^2+^ required for phosphorylation, the phosphorylation site functions in posttranslational modification, and the dimerization interface site (polypeptide binding site) allows homodimerization, which enhances binding to the target DNA [39]. Based on the above results, the transposon-inserted gene in *X. axonopodis* pv. *citri* strain TPH1 encodes for a two-component response regulator.

### BfdR is involved in *X. axonopodis* pv. *citri* biofilm formation in polystyrene microplates and on the leaf surfaces of citrus plants

**Figure 3:**
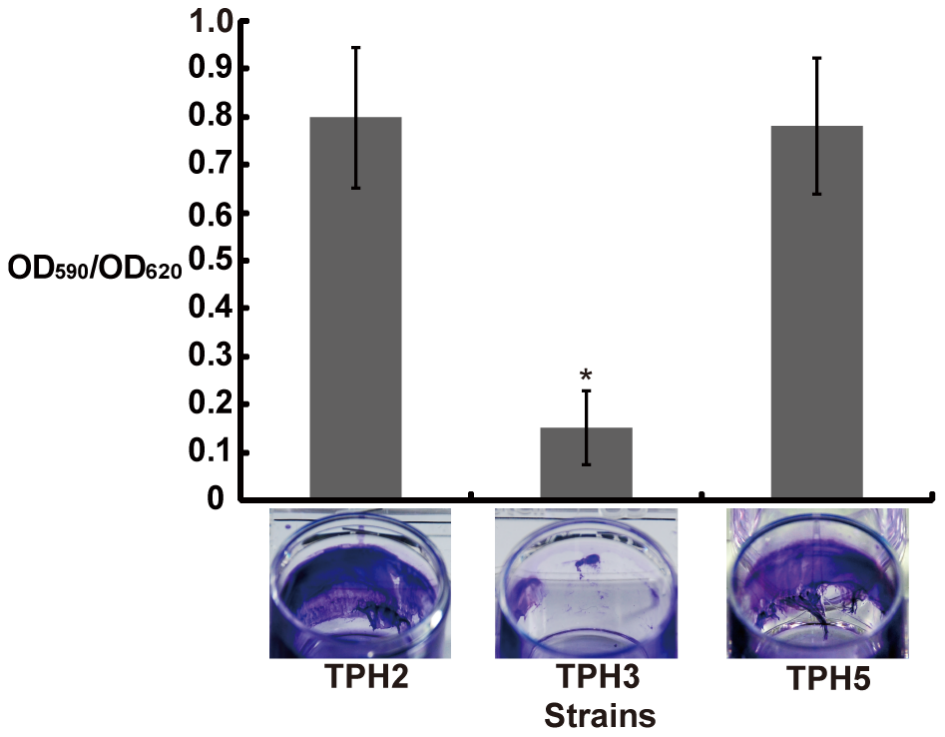
*Xanthomonas axonopodis* pv.*citri* biofilm formation in a 24-well polystyrene microplate. Experiments were performed three times with six replicates for each strain. The data presented are the means and standard deviations (error bars) from one representative experiment. *, significantly different (p<0.05) from strain TPH2 based on one-way ANOVA and Tukey's HSD test.

The mutant strain TPH1 exhibited significantly reduced biofilm formation on polystyrene plates compared with the wild type (data not shown). To determine whether deficient biofilm formation by *X. axonopodis* pv. *citri* strain TPH1 is due to a mutation in *bfdR*, *bfdR* and its predicted promoter were cloned into pBBR1MCS5 to generate pbfdR for complementation. Because the second and third start codons of BfdR and the first and second stop codons of BfdS are overlapping and the transposon is inserted in the second stop codon of BfdS (Figure 1), transposon insertion may also cause inactivation of BfdS in *X. axonopodis* pv. *citri* strain XW19. Thus, both *bfdS* and *bfdR* as well as their predicted promoters were cloned into pBBR1MCS5 to generate pbfdSR for complementation. The plasmids were electroporated into wild type and TPH1 to generate the respective complemented strains as listed in Table 1. Providing either pbfdSR or pbfdR *in trans* in strain TPH1 restored the phenotype to that of the wild type (data not shown and Figure 3, respectively). Transformation of wild type (strain TPH2) or TPH1 (strain TPH3) with the empty vector pBBR1MCS5 did not affect the phenotypes of either strains (data not shown).

**Figure 4:**
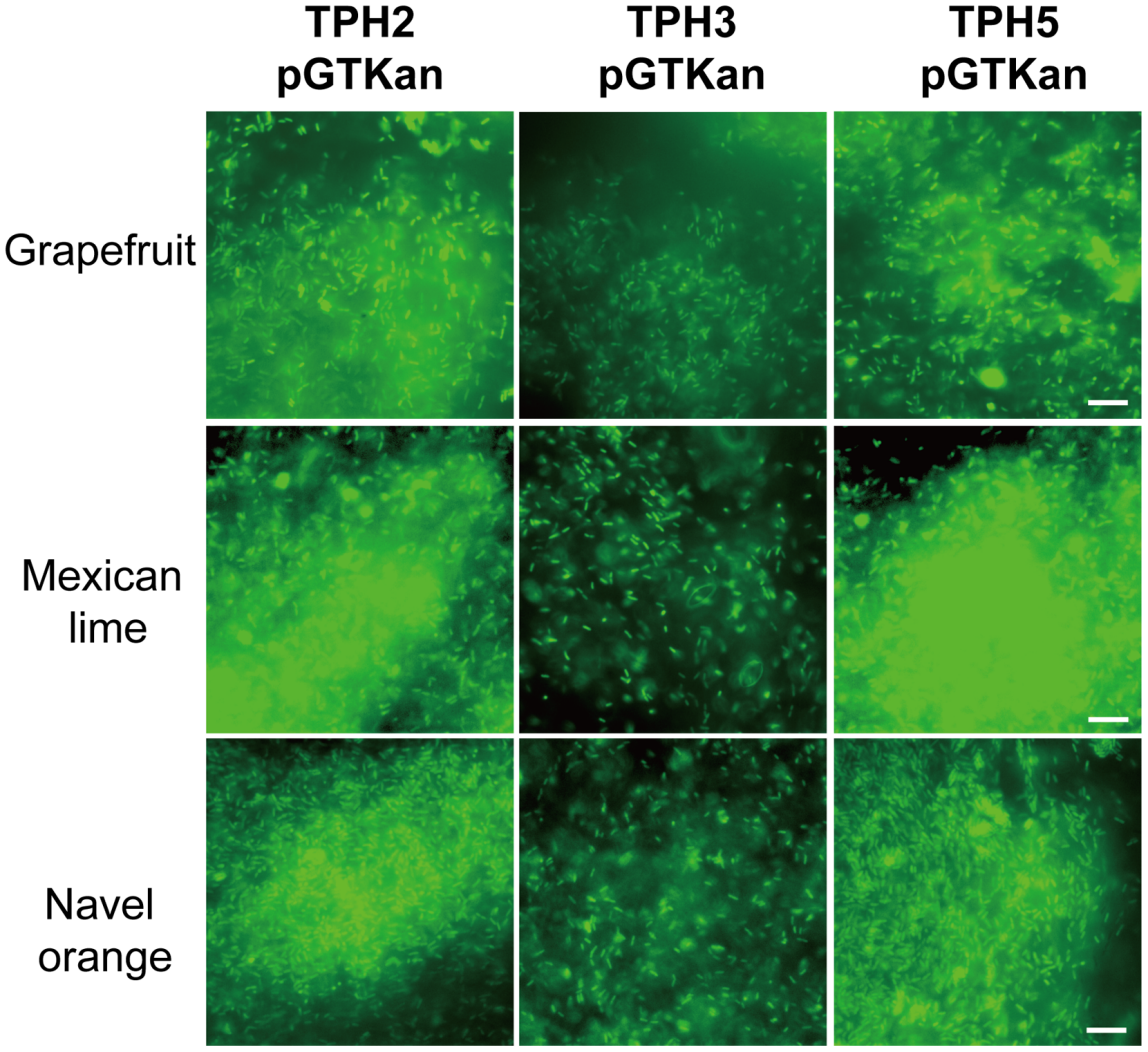
Epifluorescence micrographs of *Xanthomonas axonopodis* pv.*citri* biofilms on grapefruit, Mexican lime and navel orange leaf discs. *X. axonopodis* pv. *citri* TPH2, TPH3 and TPH5 were tagged with green fluorescent protein and expressed using the plasmid pGTKan. The culture suspensions (OD_620_  = 0.05) were inoculated in a 24-well polystyrene plate containing grapefruit, Mexican lime and navel orange leaf discs and incubated at 27°C with shaking at 50 rpm for 2 days. Scale bars, 10 μm.

**Figure 5:**
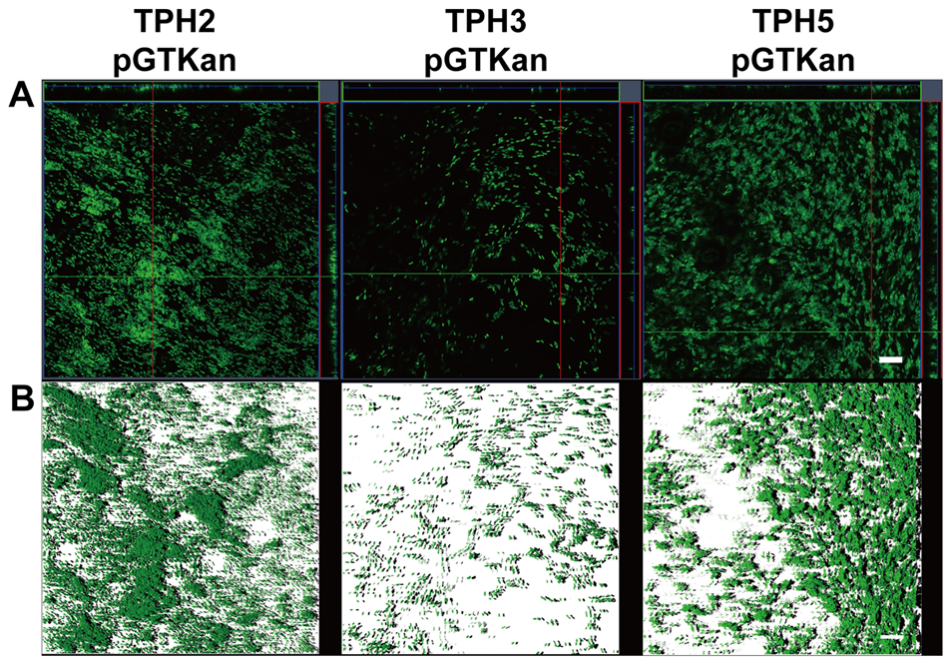
Confocal laser scanning micrographs of biofilms on Mexican lime leaf discs. *X*. *axonopodis* pv. *citri* TPH2, TPH3 and TPH5 were tagged with green fluorescent protein and expressed using the plasmid pGTKan. The culture suspensions (OD_620_  = 0.05) were inoculated in a 24-well polystyrene plate containing Mexican lime leaf discs and incubated at 27°C with shaking at 50 rpm for 2 days. **A**, Horizontal (xy-axis) biofilm section with lines indicating the positions of the xz-axis and yz-axis shown at the top and right margins of the images, respectively. **B**, A simulated projection shows a field of 141.56 μm ×141.56 μm ×9.55 μm (xyz) in TPH2/pGTKan, 141.56 μm ×141.56 μm ×9.18 μm (xyz) in TPH3/pGTKan and 141.56 μm ×141.56 μm ×10.65 μm (xyz) in TPH5/pGTKan. Scale bars, 10 μm.

Epifluorescence microscopy was used for observation of biofilms produced on the leaf surfaces of citrus plants by *X. axonopodis* pv. *citri* strains. Biofilm formation by the *bfdR* mutant (TPH3/pGTKan) was significantly decreased on the leaf surfaces of grapefruit, Mexican lime, and navel orange compared with the wild type (TPH2/pGTKan) and the complemented strain (TPH5/pGTKan) (Figure 4). Additionally, the cells of the wild type (TPH2/pGTKan) and the complemented strain (TPH5/pTGKan) were observed to be clustered together, forming microcolonies and biofilm (Figure 5). The thickness of the biofilm was approximately 6 µm. However, only a few cells of the *bfdR* mutant (TPH3/pTGKan) were clustered, and the thickness of the biofilm was approximately 1 µm. These results indicate that BfdR is involved in *X. axonopodis* pv. *citri* biofilm formation on abiotic surfaces (polystyrene microplates) as well as biotic surfaces (citrus leaf surfaces).

### A mutation in BfdR affects the epiphytic growth of *X. axonopodis* pv. *citri* on the leaf surfaces of Mexican lime

**Figure 6:**
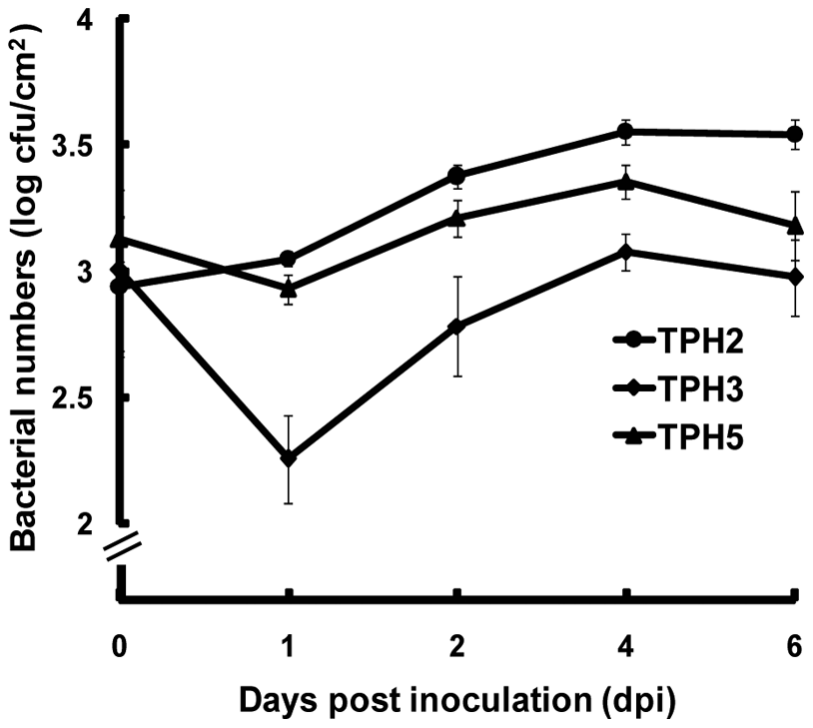
Epiphytic growth of *Xanthomonas axonopodis* pv.*citri* on Mexican lime leaves. Bacterial suspensions (OD_620_  = 0.3) were sprayed on the leaves of Mexican lime plants in a greenhouse. Bacterial populations were determined by homogenizing the leaves in Milli-Q water followed by dilution and plating at 0, 1, 2, 4, and 6 days post-inoculation. All experiments were performed three times with similar results. The results shown are the means and standard deviations (error bars) of triplicates from one representative experiment.

Previous results from Rigano *et al*. (2007) indicate that biofilm formation is important for epiphytic survival and canker development in *X. axonopodis* pv. *citri* [5]. To evaluate the role of *X. axonopodis* pv. *citri* BfdR in epiphytic growth, the population density of the *bfdR* mutant (TPH3), wild type (TPH2) and the complemented strain (TPH5) were quantified in Mexican lime leaves. At all times examined, the number of *bfdR* mutant bacteria recovered from inoculated leaves was significantly lower than the numbers of wild type and complemented bacteria (Figure 6). The number of *bfdR* mutant bacteria isolated from inoculated leaves was especially low at one day post-inoculation; specifically, 1.6±1.1×10^4^ cfu/cm^2^ (2.25±0.17 log cfu/cm^2^) and 0.9±1.3×10^6^ cfu/cm^2^ (3.00±0.31 log cfu/cm^2^) *bfdR* mutant and wild type bacteria were isolated from leaves, respectively, at one day post-inoculation. At six days post-inoculation, 5.7±2.0×10^5^ cfu/cm^2^ (3.07±0.07 log cfu/cm^2^) and 4.5±1.1×10^6^ cfu/cm^2^ (3.54±0.05 log cfu/cm^2^) *bfdR* mutant and wild type bacteria were isolated from leaves. For the complemented strain TPH5, the number of bacteria recovered at one day post-inoculation (2.8±0.7×10^5^ cfu/cm^2^  = 2.92±0.05 log cfu/cm^2^) was slightly lower than the number of bacteria in the initial inoculum. At four days post-inoculation, 1.9±0.5×10^6^ cfu/cm^2^ (3.35±0.06 log cfu/cm^2^) TPH5 bacteria were recovered from leaves. The number of bacteria recovered from leaves inoculated with the complemented strain was lower than the number of wild type bacteria recovered at all times examined, which may have occurred because the plasmid pbfdR was provided *in trans* of the *bfdR* mutant or may be due to the loss of antibiotic selection *in planta*. These results suggest the BfdR plays an important role in regulating the epiphytic growth of *X. axonopodis* pv. *citri*.

### BfdR is involved in regulating canker development in *X. axonopodis* pv. *citri*

**Figure 7:**
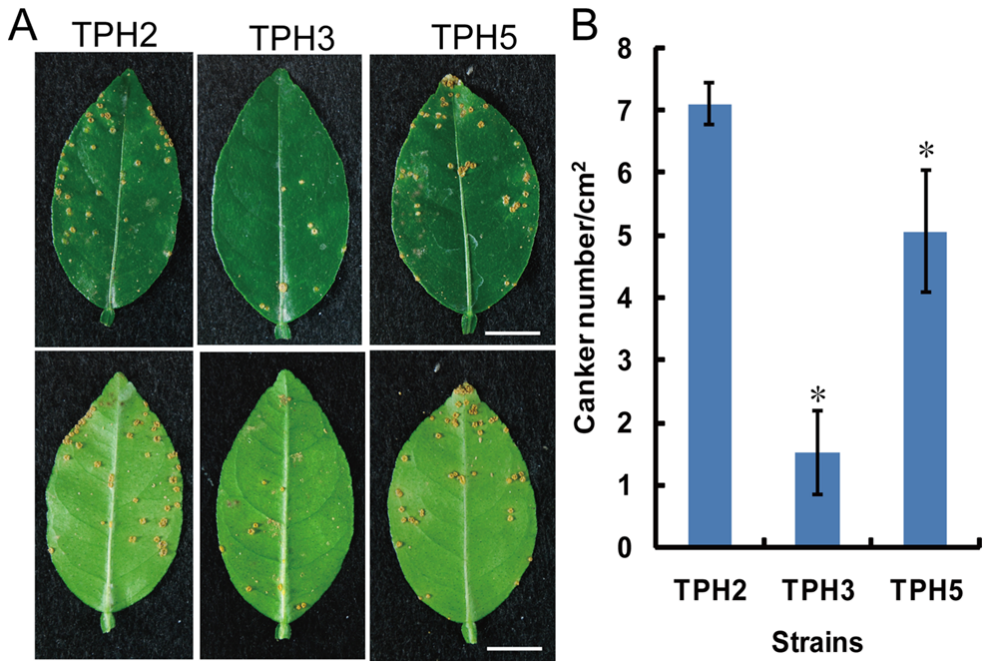
The two-component response regulator BfdR plays a role in the virulence of ***Xanthomonas axonopodis*** pv.*citri* in Mexican lime plants. **A**, Symptoms on the upper (top panel) and lower (bottom panel) leaf surfaces of Mexican lime leaves at two months post-inoculation with strains TPH2, TPH3 and TPH5. Bacterial suspensions (OD_620_  = 0.3) were inoculated on leaf surfaces using the spray method. **B**, Number of cankers per cm^2^ on each leaf. All experiments were performed three times with similar results. The results are given as the means and standard deviations (error bars) of six replicates from one representative experiment. *, significantly different (p<0.05) from strain TPH2 based on one-way ANOVA and Tukey's HSD test. Scale bars, 1 cm.

To determine whether BfdR plays a role in symptom development and virulence, TPH2, TPH3 and TPH5 strains were artificially inoculated on the leaves of Mexican lime plants. After spray inoculation, the number of cankers that had developed on both sides of the leaves inoculated with the *bfdR* mutant (TPH3) was 4.6 times lower than that observed on leaves inoculated with wild type (Figure 7) at two months post-inoculation. Complementation partially restored the phenotype of the wild type bacteria. These results indicate that BfdR plays a role in canker development in *X. axonopodis* pv. *citri* strain XW19. However, if the strains were applied by wound-inoculation, no significant difference in canker development was observed among TPH2, TPH3 and TPH5 strains. All inoculated leaves showed yellowing and developed necrotic lesions at two weeks post-inoculation. At five weeks post-inoculation, typical erumpent canker lesions with water soaked margins were visible, and the disease incidence rate reached 100% (data not shown). These data suggest that BfdR may be involved in the early stages of leaf surface colonization.

### BfdR positively regulates the transcription of *rpfF* in *X. axonopodis* pv. *citri*

**Figure 8:**
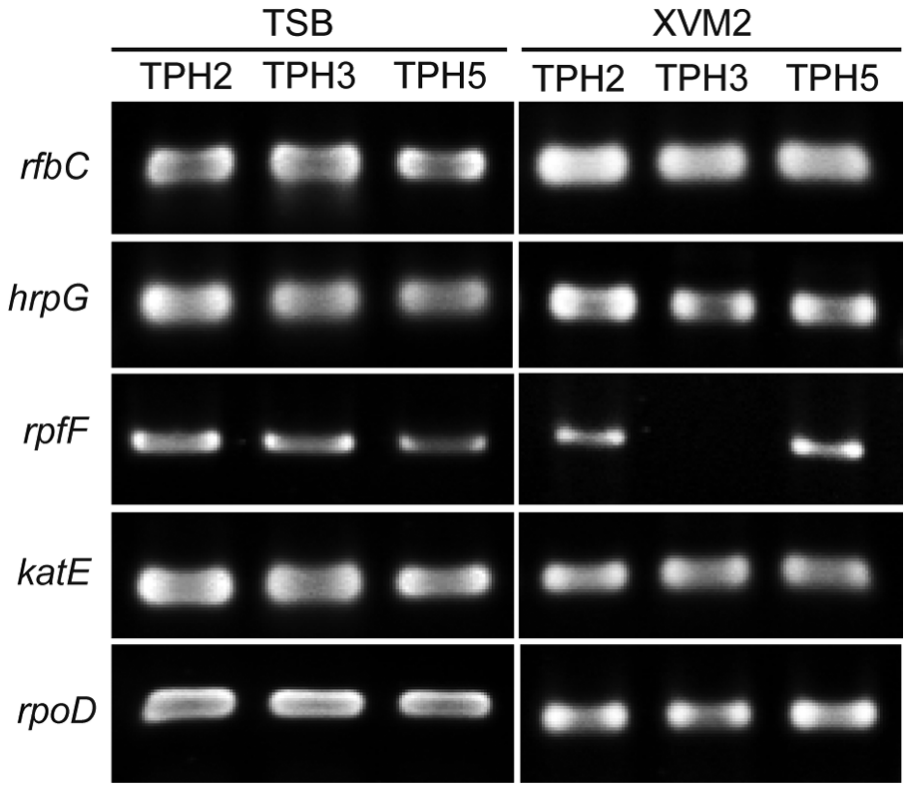
Virulence-related gene expression in Xanthomonas *axonopodis* pv.*citri* measured by RT-PCR analysis. RNA was isolated from cultures of strains TPH2, TPH3 and TPH5 in TSB or XVM2 medium, the latter of which mimics cytoplasmic fluids *in planta*, at 27°C for 18 hr with shaking at 100 rpm. RT-PCR was performed with primers specific for *rfbC* (113 bp), *hrpG* (747 bp), *rpfF* (870 bp), *katE* (127 bp) and *rpoD* (263 bp). The mRNA level of *rpoD* was used as loading control. The experiments were performed three times with similar results, and representative results from one experiment are shown.

Our results showed that symptom development and virulence are controlled by BfdR in *X. axonopodis* pv. *citri* strain XW19. We performed RT-PCR to determine whether the regulation of symptom development and virulence by BfdR is associated with expression regulation of virulence-related genes. We examined gene expression levels in TS broth and XVM2 medium, which mimics cytoplasmic fluids *in planta* [11]. Our results showed that the expression of *rpfF*, which is required for the biosynthesis of a diffusible signal factor, was upregulated by BfdR in XVM2 medium but not in TS broth after an 18 hr incubation period (Figure 8). However, the expression levels of genes involved in the synthesis of LPS O-antigen (*rfbC*), key regulator of type III secretion system (*hrpG*), and catalase (*katE*) were not affected by the mutation in *bfdR* in TS broth or XVM2 medium. *rpoD*, which encodes for sigma factor 70, was constitutively expressed in both TS broth and XVM2 medium and was used as a loading control.

Known pathogenicity factors in *Xanthomonas* include *r*egulation of *p*athogenicity *f*actor (*rpf*) [40]; xanthan [40], [41]; LPS [41]; extracellular enzymes such as esterase [42], mannanase [26], endoglucanases [41], and protease [43]; PthA [44]; HrpX [20], [41]; and catalase [45]. To understand whether a mutation in *X. axonopodis* pv. *citri* strain XW19 BfdR affects the production of extracellular enzymes or the activities of amylase, lipase, lecithinase and protease, we assessed the *bfdR* mutant and complemented strains. We found that the *bfdR* mutant had similar amylase, lipase, lecithinase, and protease activities when compared with the wild type and complemented strains (Suppl. Figure S1).

## Discussion

The TCS consists of a histidine kinase (HK) and a response regulator (RR) and plays a major role in a prokaryote's ability to sense and respond to environmental stimuli [39]. Although each *Xanthomonas* genome contains approximately 92–121 TCS genes, the biological functions of the majority of these TCS genes remain unknown [46]. In genome of *X. axonopodis* pv. *citri* strain 306, there are 35 orthodox HKs, 21 hybrid HKs, and 58 RRs [46]. In *X. axonopodis* pv. *citri* XW19, we identified a histidine kinase (BfdS) flanking BfdR, and we found that this kinase possesses a conserved asparagine at position four downstream from histidine (unpublished data); thus, BfdS is classified as a group II HK. The BfdR in *X*. *axonopodis* pv. *citri* strain XW19 shares 100% identity with locus tag XAC1284 in *X*. *axonopodis* pv. *citri* strain 306 and 89% identity with both locus tag XVE_1034 (which is a response regulator in *X. vesicatoria* strain ATCC 35937 with CheY-like receiver, ATPase and DNA-binding domains) and locus tag XCC1187 (which is a two-component regulatory protein in *X. campestris* pv. *campestris* strain ATCC33913). BfdR in *X*. *axonopodis* pv. *citri* strain XW19 contains a conserved REC domain including active sites, a phosphorylation site, and dimerization sites similar to *Escherichia coli* CheY, which is involved in direction switching in the flagellar motor [47], [48], and OmpR, which controls expression of outer membrane proteins in response to osmotic stress [49], [50]. BfdR is also similar to *Pseudomonas aeruginosa* PhoB [51]. In addition, the phosphorylated OmpR and PhoB homologues usually function to stimulate the transcription of many genes in *E. coli* and are essential for virulence or biofilm formation in numerous pathogens [52], [53], [54], [55]. However, in contrast to *E. coli* CheY [56], a mutation in *bfdR* of *X. axonopodis* pv. *citri* strain XW19 did not affect swimming or swarming motility (unpublished data).

TCSs in *X. axonopodis* pv. *citri* have been functionally characterized including RpfC/RpfG [57], HrpG [20] and ColR/ColS [21]. RpfC is a hybrid protein consisting of N-terminal transmembrane, histidine kinase, response-regulator and C-terminal histidine phosphotransfer domains that interacts with both RpfG and RpfF [57]. RpfG consists of an N-terminal REC domain and a C-terminal HD-GYP domain, the latter of which was demonstrated to exhibit 3,5-cyclic diguanylic acid (c-di-GMP) phosphodiesterase activity [57], [58]. Furthermore, RpfG was shown to interact with diguanylate cyclase GGDEF domain-containing proteins, which are responsible for the production of c-di-GMP. C-di-GMP is an important second messenger that was shown to regulate an array of bacterial processes including biofilm formation, virulence and motility [57], [59]. The OmpR family regulators HrpG and ColR contain REC domains at their N-termini and DNA-binding motifs at their C-termini [20], [21]. In contrast, BfdR in *X*. *axonopodis* pv. *citri* strain XW19 contains only the REC domain. The function of a BfdR homologue (XAC1284) in *X. axonopodis* pv. *citri* strain 306 has not been reported. Here, we have demonstrated the involvement of *X*. *axonopodis* pv. *citri* BfdR in biofilm formation and virulence and showed that BfdR positively regulates *rpfF*.

HrpG, ColR, and RpfG in *X. axonopodis* pv. *citri* were shown to play roles in coordinating the expression of multiple genes that are critical for pathogenicity [20], [21], [57]. Based on microarray analyses, HrpG was shown to regulate 24 type III secretion system genes, 23 type III secretion system effector genes, and 29 type II secretion system substrate genes in addition to genes related to chemotaxis, flagellar biosynthesis, and transport as well as regulatory genes such as *rpfG*, *flgM* (encodes for a flagellar protein), *phoU* (encodes for a phosphate regulon transcriptional regulator), and *regS* (a two-component system sensor) [20]. ColR is thought to play multiple roles in the pathogenicity of citrus canker bacteria [21]. It regulates not only virulence but also growth *in planta*, biofilm formation, catalase activity, LPS production, and resistance to environmental stress [21]. Based on quantitative RT-PCR assays, ColR positively regulated the expression of virulence-related genes including components of the type III secretion system (*hrpD6* and *hpaF*), the type III secretion system effector gene *pthA*, the LPS O-antigen synthesis gene *rfbC*, and the catalase gene *katE* [21]. Mutations in *rpfG* of *X. axonopodis* pv. *citri* were shown to reduce endoglucanase and protease activities, the production of cyclic β-(1,2)-glucan and xanthan, bacterial motility and attachment to the surface of Duncan grapefruit leaves and virulence on lemon leaves in addition to increasing the level of DSF [16], [60]. In this study, we identified a novel two-component system response regulator, BfdR, in *X. axonopodis* pv. *citri* strain XW19 and demonstrated its involvement in biofilm formation on the leaf surfaces of citrus plants, epiphytic growth, and canker development as well as its ability to regulate the expression of *rpfF*. Mutation of *bfdR* in *X. axonopodis* pv. *citri* strain XW19 did not affect amylase, protease, lipase or lecithinase activities. Semi-quantitative RT-PCR analysis indicated that the transcript levels of *rfbC*, *hrpG* (encodes for a master regulator of type III secretion system components), *katE* (encodes for catalase) were similar in the *bfdR* mutant (TPH3), the wild type (TPH2), and the complemented strain (TPH5) in both TSB and XVM2 media. However, the transcript level of *rpfF* was reduced in the *bfdR* mutant compared with the wild type in XVM2 medium. In the complemented strain, the *rpfF* transcript was restored back to wild type levels. These results suggest that BfdR in *X. axonopodis* pv. *citri* XW19 was not involved in the synthesis of known virulence-associated extracellular enzymes (including amylase, protease, lipase, or lecithinase) or genes including *rfbC*, *hrpG* and *katE*; however, BfdR positively regulated the transcription of *rpfF*. Our results showed that the expression of *rpfF* was only regulated by BfdR in XVM2 medium and not in TS broth. It is plausible that *rpfF* may be differentially expressed in XVM2 medium and TS broth. Similarly, data from Astua-Monge *et al*. (2005) suggested that the expression levels of *rpfG* and *rpfC* were decreased in XVM2 medium compared with their levels in nutrient broth [11].

RpfF encodes for an enoyl CoA hydratase and is partially dependent on RpfB, a long chain fatty acyl CoA ligase, for the synthesis of DSF [61]. Comparative genomic analyses have revealed that the *rpf* gene cluster is found in plant and human pathogens closely related to *X. campestris* including *X*. *axonopodis* pv. *citri*, *Xyllela fastidiosa*, *S. maltophila*, and *Burkholderia cenocepacia* [17], [62], [63], [64], [65]. *rpf*/DSF signaling has been shown to contribute to virulence, biofilm formation, interaction with insect vectors or/and antibiotic tolerance in these pathogens [26], [64], [66], [67], [68]. The findings in *X. campestris* and *B. cenocepacia* suggest that the two-component regulator RpfG (with HD-GYP domain) and cis-2-dodecenoic acid receptor RpfR, respectively, link the *rpf*/DSF quorum sensing system with virulence regulation via c-di-GMP turnover [69], [70]. At high cell densities, RpfC binds to DSF and phosphorylates RpfG, leading to phosphodiesterase activation and a decrease in c-di-GMP levels in *X. campestris* [58], [71]. The decrease in c-di-GMP levels activates the cNMP-binding transcription regulator Clp, which induces the expression of virulence-related genes such as those involved in the synthesis of EPS, extracellular enzymes, membrane proteins, flagella, and components of the Hrp system; iron uptake; multidrug resistance; detoxification; and biofilm dispersal [72]. Here, we identified a two-component regulator, BfdR, in *X. axonopodis* pv. *citri* XW19 that is located far from the *rpf* gene cluster. We have shown that BfdR regulates *rpfF* expression, virulence and biofilm formation. These results suggest that BfdR may be connected to virulence and biofilm formation in *X. axonopodis* pv. *citri* XW19 through the regulation network of *rpf*/DSF. It remains to be determined whether the BfdR regulation network is also linked with c-di-GMP. Additionally, the results from the wound-inoculation pathogenicity assay indicated no significant differences in canker development between the wild type strain and the *bfdR* mutant, suggesting that BfdR may be involved in the early stages of leaf surface colonization.

In conclusion, our results demonstrate that BfdR in *X. axonopodis* pv. *citri* XW19 plays a critical role in colonization, biofilm formation and virulence on the leaf surfaces of citrus plants. BfdR did not regulate the production of virulence-related extracellular enzymes including amylase, lipase, protease and lecithinase or the expression of genes involved in the synthesis of type III secretion system components, O-antigen LPS, or catalase. However, BfdR controlled the expression of *rpfF*, a gene involved in DSF synthesis, in a medium that mimics cytoplasmic fluids *in planta.*

## Supporting Information

Figure S1 ***Xanthomonas axonopodis*** **pv.** ***citri*** **wild type,** ***bfdR*** **mutant and complemented strains showed similar activities of amylase, lipase and lecithinase.** 10 μl bacterial suspensions of *X. axonopodis* pv. *citri* strains XW19, TPH1, TPH2, TPH3, TPH4 and TPH5 (OD_620_ = 0.3) were spotted on medium as the sequence shown in (**A**). Activities of extracellular enzymes for amylase (**B**), lipase (**C**), and lecithinase (**D**) by the strains were shown.
(TIF)
