# Supplementary figures and images for: A Novel Two-Component Response Regulator Links rpf with Biofilm Formation and Virulence of Xanthomonas axonopodis pv. citri
Source: PLoS One. 2013 Apr 23;8(4):e62824. doi: 10.1371/journal.pone.0062824 (PMC3633832; doi:10.1371/journal.pone.0062824)

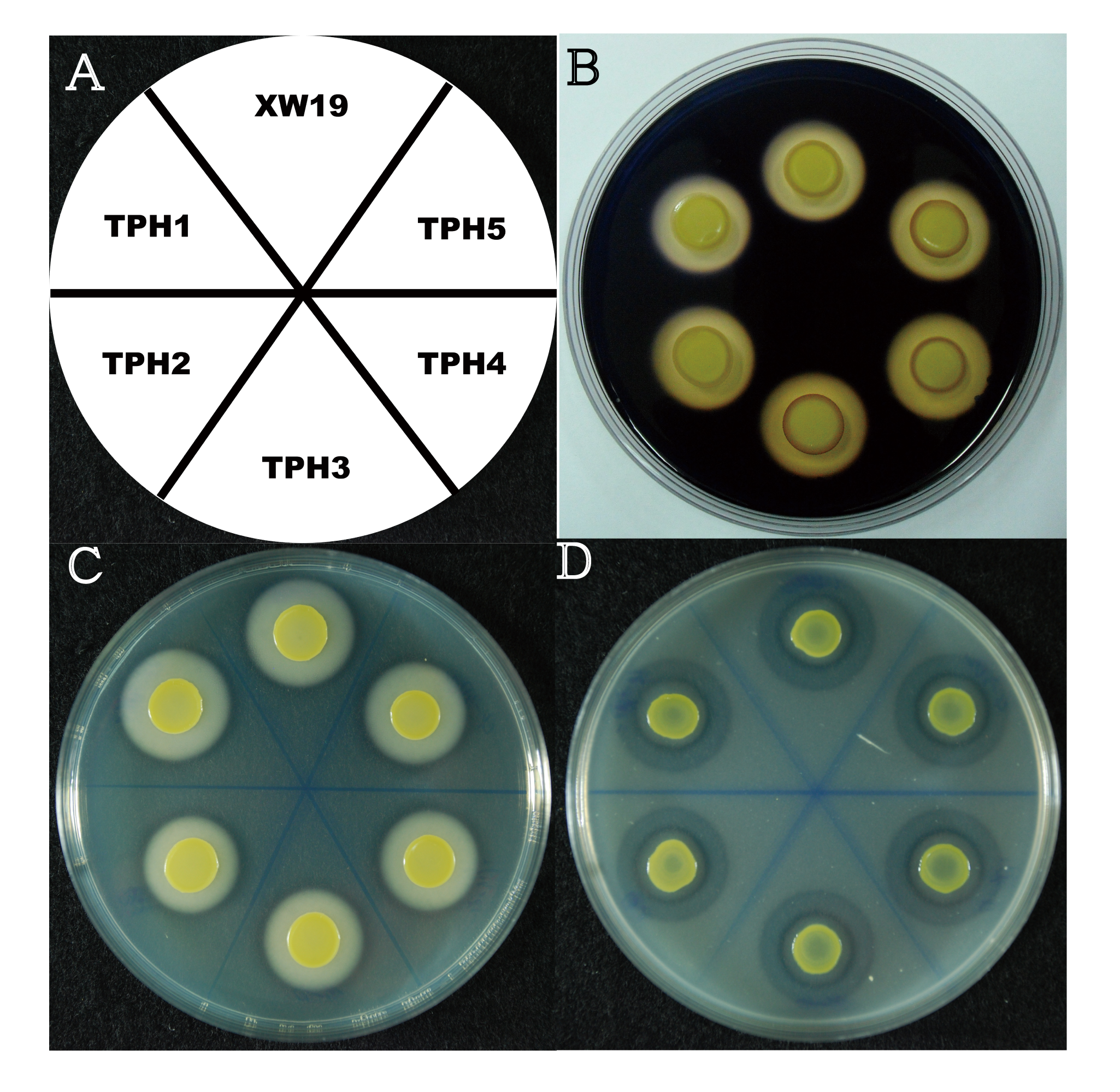

Supplement: Figure S1 — Xanthomonas axonopodis pv. citri wild type, bfdR mutant and complemented strains showed similar activities of amylase, lipase and lecithinase. 10 μl bacterial suspensions of X. axonopodis pv. citri strains XW19, TPH1, TPH2, TPH3, TPH4 and TPH5 (OD620 = 0.3) were spotted on medium as the sequence shown in (A). Activities of extracellular enzymes for amylase (B), lipase (C), and lecithinase (D) by the strains were shown. (TIF) [file pone.0062824.s001.tif]
